# Editorial: Pathogenesis of Fungal Biofilms in Different Environmental Conditions and Clinical Outcomes

**DOI:** 10.3389/fcimb.2021.778458

**Published:** 2021-10-21

**Authors:** Regina Helena Pires, Luis R. Martinez, Maria José Soares Mendes-Giannini, Maria Aparecida Resende Stoianoff

**Affiliations:** ^1^ Department of Health Promotion, University of Franca, Franca, Brazil; ^2^ Department of Oral Biology, University of Florida, Gainesville, FL, United States; ^3^ Department of Clinical Analysis, São Paulo State University, Araraquara, Brazil; ^4^ Department of Microbiology, Federal University of Minas Gerais, Belo Horizonte, Brazil

**Keywords:** biofilms, fungal pathogens, environmental factors, clinical outcome, virulence

The use of immunosuppressive drugs, broad-spectrum antimicrobials, anticancer chemotherapies, organ transplants, an aging patient population, and the intervention of hospitalized patients with numerous medical devices contribute to compromised immunity, creating an opportunistic possibility for disease by medically important fungi. Fungal infections represent a public health problem due to the synergism of the aforementioned factors and the ability of the fungus to adapt to new environmental conditions, which depends on the expression of specific virulence factors. For instance, the ability to produce specific hydrolytic enzymes, the overexpression of genes that regulate the efflux pumps activity increasing tolerance to antifungal drugs, the ability to alter cell morphology, the production of adhesins facilitating their attachment to surfaces, and the capability of forming biofilms in living tissues or inanimate substrates (e.g., central venous catheters, urinary catheters, prosthetic valves, left ventricular assist devices, hemodialysis machines, and other medical devices), are some structural or molecular factors that enhance the pathogenic potential of fungi.

Biofilms are structures formed by microbial communities that develop amidst a matrix constituted by extracellular substances, where groups of microorganisms arranged in microcolonies show high resistance to chemical disinfection, antimicrobial therapy, and a high evasion capacity against immune system response. These microbial communities can also serve as protection for the development, nutrition, metabolic cooperation, and acquisition of new genetic characteristics by the participating microorganisms, generating clinical, therapeutic, and economic implications. Among the broad range of yeast and filamentous fungi that form biofilms, *Candida* spp., *Trischosporon* spp., *Aspergillus* spp., *Paracoccidioides brasiliensis*, and *Fusarium* spp., have been identified by emerging technologies, as fungal pathogens.

From the cell’s attachment to the development of a three-dimensional structure, the growth of a biofilm is correlated with molecular aspects such as transcriptional regulatory genes that contribute to cell differentiation and adaptation to environmental cues. However, classifying the roles of all these genes is a highly complex task since the regulatory processes of biofilm formation are cyclical and dynamic.

Disease-related biofilms can be multi-species or even multi-kingdom. It has been suggested that hyphae of filamentous fungi or the filament form (pseudohyphae) of yeast provide the architecture for adhesion and development of bacterial biofilm, allowing the formation of polymicrobial biofilms. These biofilms represent a problem of significant clinical relevance since they a reserve for a wide variety of microorganisms, including fungi and bacteria. The development of polymicrobial biofilms can modify the virulence of microorganisms that are part of these interkingdom communities and the standardization of treatments that are usually effective against these pathogens, can be altered.

Due to the limited therapeutic arsenal available to treat systemic fungal infections associated with the increase in antifungal resistance and the number of infections caused by biofilms, the search for new molecules with antifungal potential is necessary. Antifungal drugs for clinical use currently comprise polyenes, flucytosine, azoles, and echinocandins, which, although effective, have a limited activity spectrum, a small number of targets, undesirable side effects, and the emergence of resistant strains. Such factors have motivated researchers around the world to develop new antifungal agents employing synthetic and semi-synthetic methodology, in addition to the isolation of a natural product with antifungal activity. Nevertheless, the emergence of resistance and the low activity of some drugs correlated to their delivery, especially in biofilms, makes it necessary to explore new strategies to develop more effective therapies.

The ability to form biofilms on both biotic and abiotic surfaces and increased tolerance and resistance to antimicrobials, including disinfectants, has contributed to the biofilm’s persistence, including fungal biofilms, in the clinical setting. Therefore, effective decontamination of these environments can significantly increase patient safety. Studies have evaluated the efficacy and compatibility with diverse applications of several clinically used disinfectants against fungal pathogens to address these challenges. However, it is difficult to develop efficacious biofilm disinfection strategies, due to the diversity of species found in multispecies communities, broad spectrum microbial susceptibility, and lack of penetration. They must also be environmentally friendly and have low toxicity to humans. Furthermore, a biphasic death pattern may appear when treated with an increasing amount of drugs whereby most of the biofilm population is killed, while a small fraction survives - persister cells. Indeed, if persister cells are isolated, regrown and repeatedly treated with high drug concentrations, the same drug response will be observed as in the original population. Once antimicrobial treatment ends, persistent ones can survive and repopulate the biofilm, being correlated with recalcitrant infections. In fact, biofilm-related infections are increasingly related to high rates of morbidity and mortality in the healthcare system and, therefore, many research efforts are needed to help with its prevention and management. In this Research Topic, a multidisciplinary team of authors who share a common interest in human fungal pathogenesis was brought together to highlight the fungal biofilm-related infections by different fungi through seven original articles and one brief communication.

The vital role of transcriptional factors in stages of biofilm formation is exemplified by the original research presented by Bitencourt et al., which emphasize the role of the APSES transcription factors family, especially StuA, in the *Trichophyton rubrum* morphogenesis and virulence. Deletion of StuA impairs biofilm formation by this dermatophyte.

The polymicrobial nature of biofilms is recognized as an essential contributor to fungal pathogenesis. This important topic was addressed in the original research by Ramírez-Granillo et al., who analyzed the aspects of the co-infection of *Aspergillus fumigatus* and *Staphylococcus aureus*, using a cell line culture model to study the impact of polymicrobial biofilms on keratitis. The complexity of fungal-bacterial interactions in polymicrobial/inter-kingdom biofilms involving dimorphic fungus was highlighted by Medina-Alarcón et al. who showed the pioneering interaction between *P. brasiliensis* and *Mycobacterium tuberculosis* as well as the possibility of treating the infection with a nanoparticle-releasing natural compound.

However, for successful management of fungal infections, the continued development of new antifungals and the appropriate use of commonly used antifungals are necessary. Evidence of the action of plant-derived products (*Cymbopogon flexuosus* essential oil and its bioactive constituent - citral) on bacterial (*Staphylococcus aureus*), fungal (*Candida* species), and mixed bacterial-fungal biofilms was provided by Gao et al. In addition, a pharmaceutical combination of the antifungal compound and innovative approaches has been used to combat fungal diseases, especially those associated with biofilms. Contribution to new antifungal biofilms strategies, combining antifungal compounds and photodynamic therapy, was provided by the original research authored by Bila et al. The antimicrobial delivery systems, a promising tool for the inactivation of fungi in biofilms, were shown by Costa-Orlandi et al. when demonstrating the comparable efficacy of nitric oxide-releasing nanoparticles on dermatophyte biofilms.

Commonly used disinfectants include halogens, peroxygens, acids, and quaternary ammonium. Biofilm resistance to disinfectants is multifactorial and results from different mechanisms, although the nature and composition of the matrix are among the most relevant factors, making antimicrobials ineffective in killing biofilm-derived fungi, even in the usable concentrations of commercial solutions. This phenomenon was addressed in the original research by Lopes et al., who demonstrated the ineffectiveness of commercial disinfectants against planktonic and biofilm cells of *Aspergillus* and *Fusarium.* The disinfectants used in the study are commonly used in hemodialysis facilities and, the most currently used – peracetic acid, was tested by exposing mice at a residual dose. Finally, Cordeiro et al. provide insights into the mechanisms that give rise to persisters cells in *Trichosporon asahii* and *T. inkin* biofilms, which can be an important finding in these species whose treatment remains controversial.

In summary, the articles in this Frontier’s topic broadly cover the spectrum of investigations on fungal biofilm composition (e.g., mono- and multi-species), host-pathogen interactions, transcription factors involved in biofilm formation regulation, environmental conditions, and new antifungal drug candidates, and provide the current state-of-the-art landscape in our understanding of biofilms by medically important fungi and relevant bacterial pathogens and warrants future studies to understand the basis of fungal biofilms biology in infection and disease.

## Author Contributions

All authors contributed to the final format of the manuscript and approved it for publication.

## Funding

National Institute of Allergy and Infectious Diseases [Martinez, LR R01-AI145559], São Paulo Research Foundation - FAPESP (Mendes-Giannini, MJS grants 2018/02785-9; Pires, RH grants 2015/19090-5) and Brazilian National Research Council – CNPq (Mendes-Giannini, MJS grants 310524/2018-0). 

## Conflict of Interest

The authors declare that the research was conducted in the absence of any commercial or financial relationships that could be construed as a potential conflict of interest.

## Publisher’s Note

All claims expressed in this article are solely those of the authors and do not necessarily represent those of their affiliated organizations, or those of the publisher, the editors and the reviewers. Any product that may be evaluated in this article, or claim that may be made by its manufacturer, is not guaranteed or endorsed by the publisher.

